# Method of Depriving Quinine of Its Bitterness

**Published:** 1850-07

**Authors:** 


*Method of depriving Quinine of its Bitterness.* By RICHARD H. THOMAS, M. D., of Baltimore.

BALTIMORE, 2d Month 15th, 1850.

TO DR. ISAAC HAYS:—

*Dear Doctor:* Believing that I have discovered a method, by which quinine may be quite deprived of its great bitterness, without injuring its virtues in the least, I take this method of making it known to the profession. Perhaps I may have been anticipated; but if it be so, I am not aware of it.

In 8th month (August last,) having occasion to prescribe for a little patient, who was affected both with diarrhœa and intermittent fever, I ordered a combination of quinine and tannic acid. The child took it so readily, that I tasted it, and was surprised to discover no taste of quinine, which I at once attributed to the combination.

I have since prescribed it in a number of instances, and found that whilst it was equally effectual, it was far more palatable than any other combination of quinine I was acquainted with. On referring to the *American Journal of the Medical Sciences*, Vol. XIX. p. 219 (1836,) it will be found that Dr. Ronander, Secretary of the Swedish Medical Association, recommended, in 1834, the tannate of quinine and cinchonin as the most active ingredients of the Peruvian bark; he asserts that he has cured by their means, several cases of obstinate ague, which had resisted the use of sulphate of quinine, and other powerful remedies, &c., &c. Nothing is said in the extract, from the original paper, in *Hecker's Annals*, December, 1834, of the taste of the tannate of quinine. Compared with the sulphate, it is almost tasteless.

The following is the extemporaneous prescription I am in the habit of ordering for a child two years old: ℞. Quiniæ sulph. gr. x; acid tannici gr. ij; aqua ℥vj; syrupa urant. ℥ij. M. A teaspoonful every hour or two.

I enclose a note on the subject, from one of our most intelligent and careful apothecaries:

BALTIMORE, February 6, 1850.

*Dear Sir:* I find, after trying a number of times, combinations of quiniæ sulphas and acidi tannici in different proportions, that ten grains may be deprived of its bitterness in a great degree, by the addition of one grain and a half of tannic acid. I think this is a proper proportion.

Respectfully, JAMES V. D. STEWART.

DR. R. H. THOMAS.

*American Journal of Medical Sciences.*

